# Effects of endurance exercise on skeletal muscle and liver metabolic health in male rats with different fitness under chronic circadian rhythm disruption

**DOI:** 10.3389/fendo.2026.1841754

**Published:** 2026-07-15

**Authors:** Yu Gu, Wenduo Liu, Zilin Wang, Sang Hyun Kim

**Affiliations:** 1 Department of Martial Arts and Traditional National Sports, Henan Sport University, Zhengzhou, China; 2 Department of Sports Science, College of Natural Science, Jeonbuk National University, Jeonju, Republic of Korea; 3 College of Physical Education, Beihua University, Jilin, China

**Keywords:** circadian rhythm, endurance exercise, fitness, metabolic health, NAFLD

## Abstract

**Background:**

Circadian rhythm (CR) disruption is a major risk factor for metabolic dysfunction in skeletal muscle and liver. Although endurance training (ETR) is known to improve metabolic health, it remains unclear whether exercise timing and training duration influences metabolic adaptations under CR disruption.

**Methods:**

Twenty-four male Sprague–Dawley rats were randomly assigned to four groups: regular sleep cycle sedentary (RSC), irregular sleep cycle sedentary (ISC), irregular sleep cycle with late-stage ETR (ISE), and irregular sleep cycle with prolonged ETR (IEE). CR disruption was induced by alternating light–dark cycles every three days for 20 weeks. ETR was performed at moderate intensity. Skeletal muscle and liver samples were analyzed for circadian clock proteins, mitochondrial biogenesis, glucose and lipid metabolism, oxidative stress, and fibrosis-related markers.

**Results:**

CR disruption significantly impaired circadian clock regulation, mitochondrial biogenesis, and metabolic function in both skeletal muscle and liver. Specifically, reductions in BMAL-1 expression, AMPK activation, PGC-1α signaling, and oxidative phosphorylation were observed, along with disrupted glucose and lipid metabolism. These alterations were accompanied by increased oxidative stress, hepatic lipid accumulation, and fibrosis-related markers. ETR effectively attenuated these changes. Notably, training initiated prior to CR disruption (or a higher fitness maintained during CR disruption) group (IEE) resulted in greater improvements in mitochondrial and metabolic adaptations compared with shorter-duration training (ISE). In contrast, no significant differences were observed between ISE and IEE in oxidative stress and fibrosis-related outcomes.

**Conclusions:**

Endurance training mitigates CR disruption–induced metabolic dysfunction in skeletal muscle and liver. Prolonged training exposure enhances mitochondrial and metabolic adaptations, whereas protective effects on oxidative stress and fibrosis may occur even with shorter training duration. These findings highlight the importance of exercise timing and training duration in optimizing metabolic resilience under circadian disruption.

## 1 Introduction

Disruption of circadian rhythm (CR), as commonly observed in shift work, irregular sleep patterns, and modern lifestyle conditions, has been increasingly recognized as a critical contributor to metabolic disorders such as insulin resistance, obesity, and non-alcoholic fatty liver disease (NAFLD) (1). CR plays a fundamental role in regulating metabolic homeostasis across multiple organs, including skeletal muscle and liver (2–4). Accumulating evidence suggests that circadian misalignment impairs mitochondrial function, alters substrate metabolism, and promotes oxidative stress, ultimately leading to systemic metabolic dysfunction (4).

Importantly, populations exposed to circadian disruption are heterogeneous in terms of habitual physical activity (5). For example, individuals with disrupted circadian rhythms may include both predominantly sedentary workers (e.g., office-based occupations) and physically active laborers (6). These groups may differ substantially in baseline physiological status and metabolic resilience. Although regular exercise is widely recognized as an effective strategy for improving metabolic health, it remains unclear whether and how differences in physical activity history influence metabolic adaptations under conditions of circadian disruption (7).

In addition, emerging evidence from various injury and stress models indicates that exercise interventions do not universally exert beneficial effects (8); rather, the timing and duration of exercise exposure may critically determine the outcome (9). In certain pathological conditions, premature or insufficiently adapted exercise may even exacerbate tissue damage, whereas appropriately timed or sustained exercise can promote recovery and adaptation (10). This suggests that the initiation timing and training history of exercise interventions may be key factors influencing their effectiveness under stress conditions, including circadian disruption.

Despite growing interest in the interaction between circadian rhythms and endurance training (ETR), accumulating evidence has confirmed that ETR improves metabolic health (11). However, the roles of exercise timing and training duration in modulating metabolic responses to chronic circadian disruption remain poorly understood. In particular, it remains unclear whether, compared with ETR initiated after CR disruption, a higher training status established prior to or during CR disruption provides additional protective benefits. Moreover, whether different metabolic systems exhibit differential sensitivity to exercise duration under circadian misalignment remains to be elucidated.

Therefore, the present study aimed to investigate the effects of ETR on skeletal muscle and hepatic metabolic health under chronic CR disruption, with a specific focus on the influence of training status and exercise duration. Using a rat model of long-term circadian disruption, we examined circadian clock regulation, mitochondrial biogenesis, substrate metabolism, oxidative stress, and fibrosis-related pathways in both skeletal muscle and liver. We hypothesized that (1) CR disruption induces coordinated metabolic dysfunction across tissues (2), ETR mitigates these alterations, and (3) Training initiated prior to CR disruption, or a higher fitness maintained during CR disruption, may enhance mitochondrial and metabolic adaptations during subsequent ETR. In contrast, pathological processes such as oxidative stress and fibrosis may exhibit differential sensitivity to exercise duration. The findings of this study may provide a theoretical basis for the application and optimization of exercise prescriptions in individuals with metabolic disorders associated with circadian rhythm disruption.

## 2 Methods

### 2.1 Animal

In this study, we used 24 male Sprague–Dawley (SD) rats obtained from DaeMul Science Inc. (Daejeon, South Korea), each aged 12 weeks (84 days of age). The rats were housed in pairs per cage, maintaining humidity (40%–60%) and a consistent temperature (18 °C–22 °C). The feed (carbohydrates 58.9%, fat 12.4%, protein 28.7%; DBL, Yongin, Korea) and water were provided ad libitum throughout the experimental period. A daily light-dark cycle was established, with the period from 8 AM to 8 PM designated as daytime.

Body weight of all rats was measured every Sunday at 09:00 a.m. throughout the experiment using an electronic balance. Fixed-time weekly weighing was performed for all animals to monitor body weight changes and eliminate time-related experimental bias.

**Figure 1:**
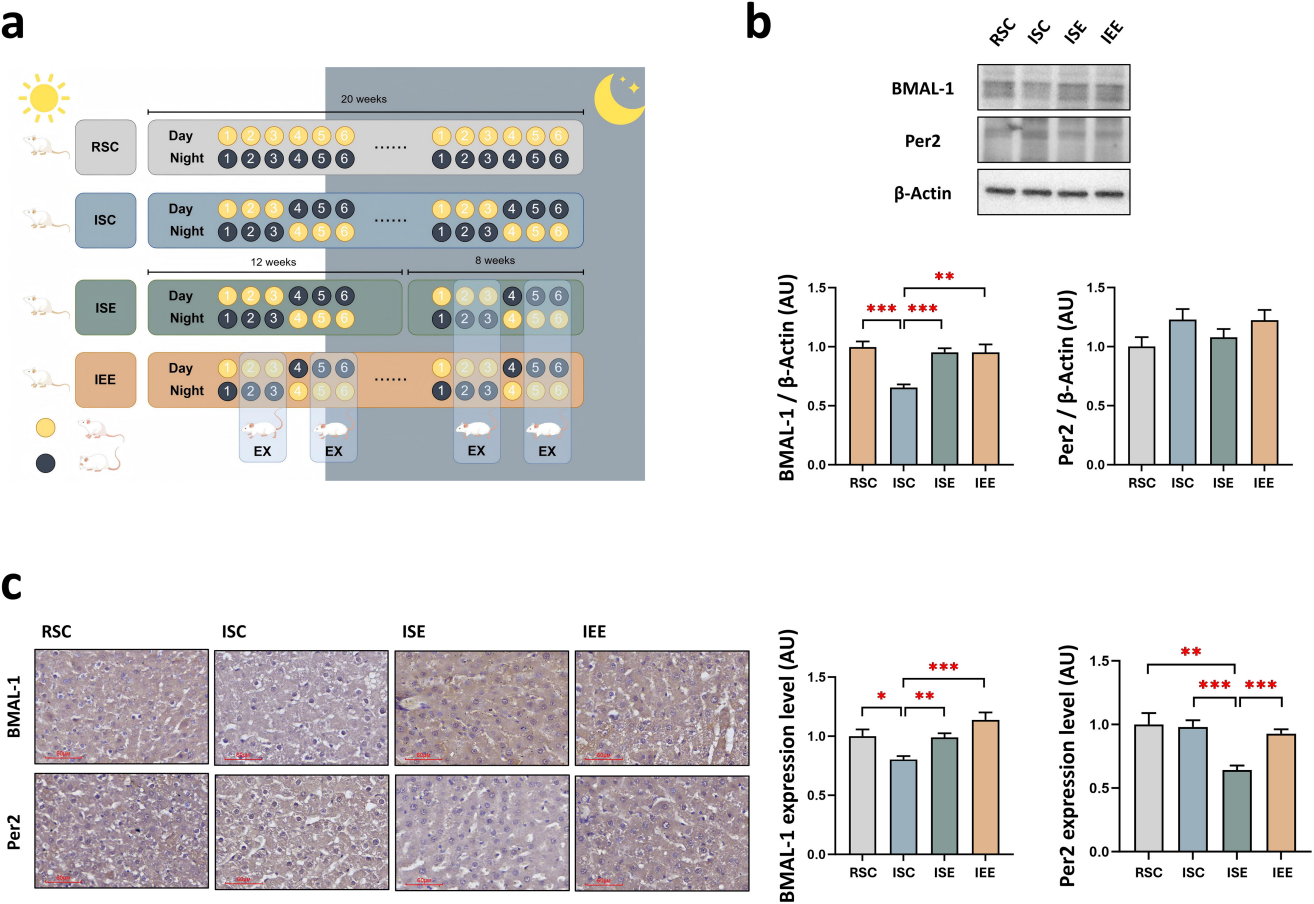
Effects of ETR on clock factor in skeletal muscle and liver of rats under chronic circadian rhythm disruption. **(A)** Research design (By Figdraw; ID: UIPUY5abeb). **(B)** Expression levels of BMAL-1 and Per2 protein in the gastrocnemius muscle as analyzed by Western blotting, along with representative images of BMAL-1, Per2 and β-actin. **(C)**Representative IHC staining and semi-quantitative analysis of BMAL-1 and Per2 immunoreactivity in the liver. Data are presented as mean ± SD (n = 6 per group). Statistical analysis was performed using one-way ANOVA followed by Tukey’s *post hoc* test for multiple comparisons (P < 0.05; *P < 0.01; **P < 0.001; ns, not significant, P > 0.05).

After two weeks of adaptive feeding, the 24 rats were numbered sequentially from 1 to 24. Then, a simple random sampling method was adopted to randomly divide all rats into four groups using a random number table, with 6 rats in each group: the 20 weeks regular sleep cycle sedentary group (RSC, n = 6), the 20 weeks irregular sleep cycle sedentary group (ISC, n = 6), 12 weeks sedentary + 8 weeks ETR (with ISC) group (ISE, n = 6), and 20 weeks ETR (with ISC) group (IEE, n = 6) (**Figure 1A**). While RSC rats were housed under a consistent light-dark cycle throughout the entirety of the experimental period, ISC rats experienced CR disruption by alternation of the light-dark cycle every three days. Specifically, the day–night light-dark rhythm was completely reversed at 8:00 a.m. on the third day of each cycle, and this circadian intervention was sustained throughout the 20-week experimental period. During the initial 12 weeks following group allocation, sleep cycles were manipulated to measure changes due to CR regulation (12). After completing the whole intervention, the SD rats were sacrificed, and tissue samples were collected for analysis.

All procedures were conducted with the approval of the Institutional Animal Care and Use Committee of Jeonbuk National University (IACUC approval no. CBNU-2022-0067).

### 2.2 Exercise protocol

During this ETR period, CR conditions were administrated in the same manner. ETR involved the use of a treadmill (TREADMILL, L.M.S. KOREA) at a fixed incline of 2%. The exercise intensity was set at approximately 60%–70% of maximum oxygen intake based on the study by Qin et al. (13). The exercise regimen was structured accordingly: a warm-up phase (10–15 m/min, 20 min), the main exercise phase (15 m/min, 30 min), and a cool-down phase (10 m/min, 10 min) for a total of 60 min. For the ISC group, exercise sessions were conducted on the second and third days of the three-day interval sleep cycle, each session lasting for a duration of one hour (07:30–08:30 am). This exercise window partially overlapped with the transition from the rest phase to the active phase under circadian disruption conditions. The timing was selected because the onset of the active phase is associated with marked endocrine fluctuations, including glucocorticoid/corticosterone-related rhythmic peaks, thereby providing a standardized physiological transition window for evaluating exercise adaptation under circadian misalignment. The treadmill exercise was modified and improved based on the method used in the previous studies (14).

### 2.3 Endurance exercise capacity test

Endurance exercise capacity (EEC) was assessed in all rats 24 h (8:30 am) after the completion of the 12-week intervention period to determine baseline fitness levels prior to the final 8 weeks endurance training phase.

The EEC test was performed using a motorized treadmill with a fixed incline of 15°. The protocol began with an initial speed of 10 m/min for 5 min, followed by incremental increases of 2 m/min every minute until exhaustion. Exhaustion was defined as the inability of the animal to maintain running despite gentle encouragement (e.g., sponge stimulation) and remaining on the electric grid for more than 3s.

Endurance performance was evaluated by recording total running time, vertical distance, and total work, which were calculated according to previously established methods (14). These parameters were used as indicators of baseline fitness capacity.

### 2.4 Sample collection

After 20 weeks treatment, the rats were anesthetized at 20 hours after the last exercise session (6:00 am) by an intraperitoneal injection of a solution consisting of Zoletil (Virbac Laboratories, Carros Cedex, France), Rompun (Bayer Korea, Seoul, Korea), and normal saline diluted at a 2:1:2 ratio (1 mL/kg body weight).

The samples were collected after anesthesia. The blood samples were drawn from the inferior vena cava. The collected blood was subject to centrifugation to isolate the serum, which was subsequently stored at –80 °C until analysis. The gastrocnemius muscle (Gas) sample were frozen rapidly in liquid nitrogen and stored at −80 °C until further analysis. The Liver samples were fixed in 10% formalin and stored at 4 °C until further analysis.

### 2.5 Western blot analysis

Gas muscle extracts were prepared, and western blotting was performed as described previously (15). The antibodies used are as follows: β-actin (Invitrogen, MA1-140, USA), AMP-activated protein kinase α1/2 (AMPKα1/2, SCBT, sc-25792, USA), phospho-AMP-activated protein kinase α1/2 (p-AMPK, Millipore, #07-681, USA), peroxisome proliferator-activated receptor-gamma coactivator-1alpha (PGC-1α; GenTex, GTX37356, USA), mitochondrial transcription factor A (mtTFA; SCBT, sc-166965, USA), oxidative phosphorylation (OXPHOS, abcam, ab110413, USA), brain and muscle ARNT-like 1 (BMAL-1; SCBT, sc-365645, USA), period circadian regulator 2 (PER2; Invitrogen, #PA5-100107, USA), cluster of differentiation 36 (CD36, SCBT,sc-7309, USA), acetyl-CoA carboxylase (ACC, Cell Signaling,#3662, USA), phospho-acetyl-CoA carboxylase (p-ACC, Cell Signaling,#3661, USA), carnitine palmitoyltransferase-1 (CPT-1, SCBT,sc-98834, USA), acyl-CoA dehydrogenases (LCAD, Abcam,ab129711, USA), glucose transporter type 4 (GLUT-4, SCBT, sc-53566, USA), hexokinase 2 (HK2; SCBT, sc-374091, USA), lactate dehydrogenase (LDH, SCBT, sc-133123, USA), mouse anti-goat (SCBT, sc-2354, USA), mouse anti-rabbit (SCBT, sc-2357, USA),and goat anti-mouse (SCBT, sc-2005, USA). Protein visualization was conducted using the ECL Western Blotting Detection Reagent (GE Healthcare, UK), and quantification was carried out using the ChemiDoc XRS+ system (BIO-RAD, USA).

### 2.6 Hematoxylin and eosin staining

Histological analysis of liver was performed according to a previously developed protocol (16). Tissues were fixed using formalin at 4 °C (After the fixed period of one week, proceed to the next steps). After dehydration with ethanol, each tissue was clarified, infiltrated, and embedded in paraffin with xylene. The embedded tissues were sectioned and stained with hematoxylin and eosin (H&E) on glass slides. The size of muscle fibers was measured using a Motic Easy Scan One slide scanner (Meyer Instruments, Inc., USA) and further quantified in ImageJ software (version 1.51, NIH, USA).

### 2.7 Immunohistochemistry

Immunohistochemistry (IHC) was conducted following the methods described previously (17). Tissue sections were stained with each of primary antibodies: BMAL-1 (SCBT, sc-365645, USA), PER2 (Invitrogen, #PA5-100107, USA), CD36 (SCBT,sc-7309, USA), ACC (Cell Signaling,#3662, USA), CPT-1 (SCBT,sc-98834, USA), Peroxisome proliferator-activated receptor-α (PPAR-α, SCBT, sc-9000, USA), PPAR-γ (SCBT, sc-7273, USA), TGF-β1 (SCBT, sc-130348, USA), Collagen 1 alpha 1 (COL1A1, SCBT, sc-293182, USA). The VECTASTAIN Universal Elite ABC Kit (Vector Laboratories, Burlingame, CA, USA) and DAB Substrate kit (Vector Laboratories) were utilized for the detection of proteins through the IHC staining. Digital images of the stained tissues were captured using the Motic Easy Scan One slide scanner (Meyer Instruments, Inc.). These images were subjected to quantitative analysis for the positive area, integrated optical density, and mean optical density using Image Pro Plus 6 (Media Cybernetics Inc., Rockville, MD, USA).

### 2.8 Thiobarbituric acid reactive substances assay

Thiobarbituric acid-reactive substances (TBARs), which are oxidative stress indicators, were analyzed after storing isolated serum at -80 °C until analysis. The supernatant was analyzed according to the manual for a Quantichrom TBARS Assay Kit (Bioassay Systems, Hayward, USA) (18).

### 2.9 Statistical analysis

All data are expressed as the mean ± standard deviation (SD) and were analyzed using GraphPad Software (Prism 10, MA, USA). One-way analysis of variance (ANOVA) was performed to analyze the body composition results for each group before and after treatment, followed by a Tukey *post-hoc* test. P < 0.05 was considered statistically significant.

## 3 Results

### 3.1 Baseline exercise capacity with chronic circadian rhythm disruption

To verify whether the 12-week CR manipulation effectively disrupted peripheral clock regulation, the expression of key clock proteins in the gastrocnemius muscle was assessed (**Supplementary Figure 1**). All tissue samples were collected at 6:00 a.m. to eliminate the interference of circadian variation on protein expression. The results showed that 12 weeks of ISC significantly reduced the expression of BMAL-1 (P < 0.001) and increased the expression of Per2 (P < 0.05) compared with the RSC group. These findings confirm that prolonged ISC induces a substantial disruption of skeletal muscle circadian clock regulation.

The effects of 12-week CR disruption on body weight and baseline exercise capacity are presented in **Supplementary Figure 2**. CR disruption resulted in a significant reduction in body weight across the ISC (P < 0.05), ISE (P < 0.01), and IEE (P < 0.01) groups compared with the RSC group. Despite reduced body weight, endurance exercise capacity differed among groups. The IEE group, which underwent exercise during the CR disruption period, exhibited significantly greater running time, vertical distance, and total work compared with the ISC and ISE groups (P < 0.01).

These results indicate that prior endurance training during CR disruption led to distinct differences in exercise capacity, thereby establishing different training status among groups before the final intervention phase.

The observed differences in exercise capacity between groups may have relevance to real-world conditions in which individuals exposed to circadian disruption exhibit varying levels of habitual physical activity. For example, shift-working populations may include both physically active occupations and predominantly sedentary roles, which could differentially influence metabolic resilience under circadian misalignment.

### 3.2 Effects of different basal fitness levels on the expression of ETR-regulated clock proteins in skeletal muscle and liver

Chronic CR disruption significantly impaired the expression of core clock proteins in both skeletal muscle and liver (**Figure 1**).

In skeletal muscle, the ISC group exhibited a significant reduction in BMAL1 protein expression compared with the other groups (**Figure 1B**; P < 0.01), whereas no significant differences in PER2 expression were observed among groups (**Figure 1B**). Both ISE and IEE groups showed restoration of BMAL1 expression relative to the ISC group.

In liver tissue, IHC staining demonstrated a similar trend toward reduced BMAL1 immunoreactivity in the ISC group (**Figure 1C**; P < 0.05). In addition, PER2 immunostaining appeared lower in the ISE group compared with the other groups (**Figure 1C**; P < 0.01). Notably, the IEE group showed partially restored staining patterns comparable to those observed in the RSC group. These findings should be interpreted as supportive histological evidence due to the semi-quantitative nature of DAB-based IHC analysis.

Overall, ETR partially or fully restored the expression of circadian clock proteins under CR disruption, with more pronounced effects observed in the IEE (High fitness) group.

### 3.3 Effects of different basal fitness levels on mitochondrial biogenesis and glucose metabolism in skeletal muscle

**Figure 2:**
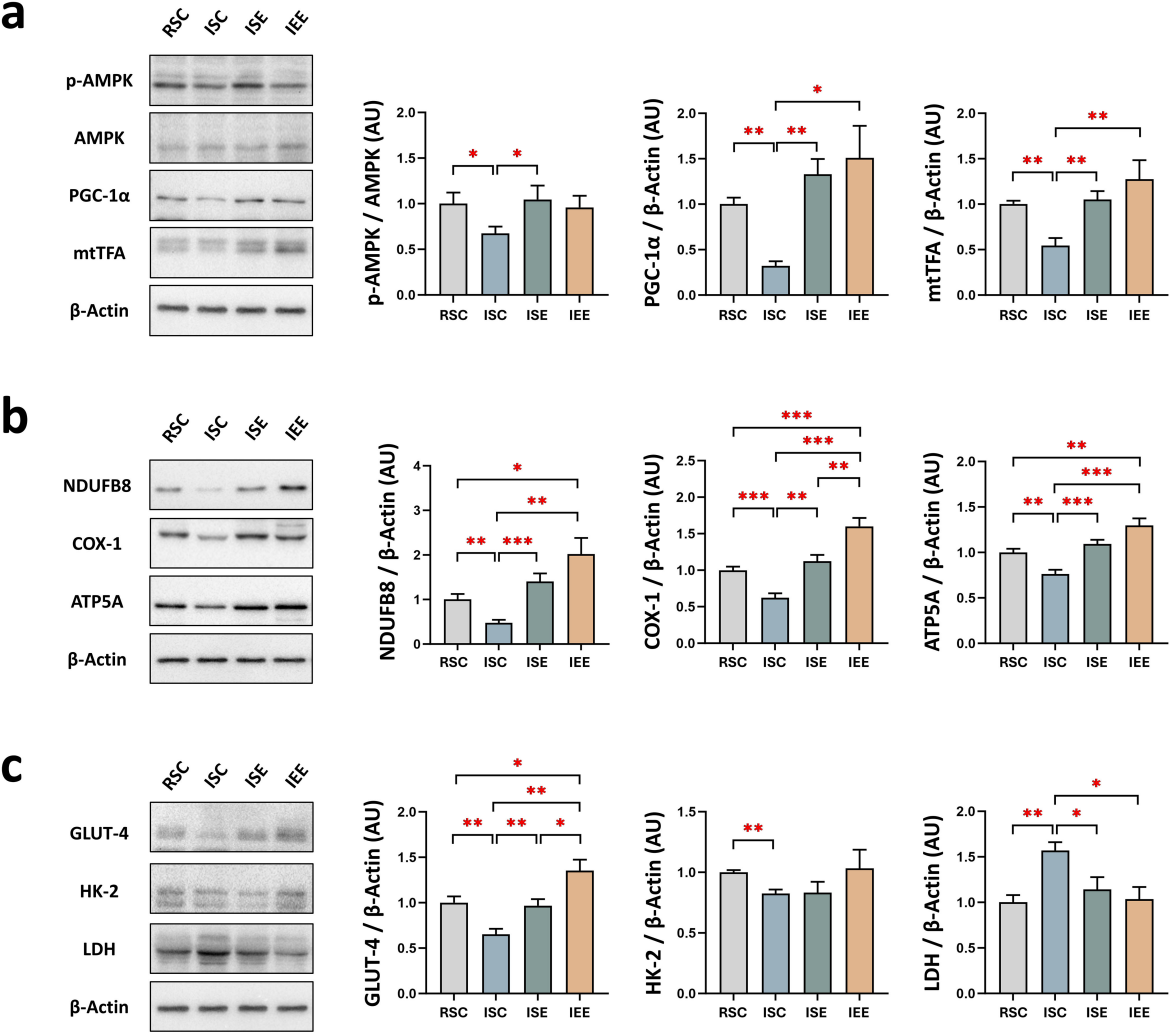
Effects of ETR on mitochondrial biogenesis and glucose metabolism–related factors in skeletal muscle of rats under chronic circadian rhythm disruption. **(A)** Expression levels of p-AMPK/AMPK ratio, PGC-1α, and mtTFA protein in the gastrocnemius muscle as analyzed by Western blotting, along with representative images of p-AMPK, AMPK, PGC-1α, mtTFA and β-actin. **(B)** Expression levels of NDUFB8, COX-1, and ATP5A protein in the gastrocnemius muscle as analyzed by Western blotting, along with representative images of NDUFB8, COX-1, ATP5A and β-actin. **(C)** Expression levels of GLUT-4, HK-2, and LDH protein in the gastrocnemius muscle as analyzed by Western blotting, along with representative images of GLUT-4, HK-2, LDH, and β-actin. Data are presented as mean ± SD (n = 6 per group). Statistical analysis was performed using one-way ANOVA followed by Tukey’s *post hoc* test for multiple comparisons (P < 0.05; *P < 0.01; **P < 0.001; ns, not significant, P > 0.05).

To evaluate the regulatory effects of ETR on CR disruption–induced metabolic dysfunction, mitochondrial biogenesis, oxidative phosphorylation, and glucose metabolism–related proteins in skeletal muscle were analyzed (**Figure 2**).

CR disruption markedly impaired mitochondrial biogenesis and glucose metabolism in skeletal muscle. Compared with the RSC group, the ISC group exhibited significantly reduced phosphorylation of AMPK (P < 0.05), along with decreased expression of PGC-1α (P < 0.01) and mtTFA (P < 0.01) (**Figure 2A**). In addition, the expression of oxidative phosphorylation (OXPHOS) complex proteins, including NDUFB8 (P < 0.01), COX-1 (P < 0.001), and ATP5A (P < 0.01), was consistently decreased (**Figure 2B**). Similarly, glucose metabolism–related proteins, including GLUT4 (P < 0.01) and HK2 (P < 0.01), were significantly downregulated in the ISC group, whereas LDH (P < 0.01) expression was relatively increased (**Figure 2C**).

ETR significantly ameliorated these alterations. Both ISE and IEE groups showed enhanced AMPK activation and increased expression of PGC-1α, mtTFA, OXPHOS complex proteins, and GLUT4 compared with the ISC group (**Figure 2**; P < 0.05). Notably, the IEE group exhibited significantly higher expression levels of COX-1 (**Figure 2B**) and GLUT4 (**Figure 2C**) than the ISE group (P < 0.05), indicating a greater extent of metabolic restoration with prolonged training exposure.

### 3.4 Effects of different basal fitness levels on the lipid metabolism in skeletal muscle and liver

**Figure 3:**
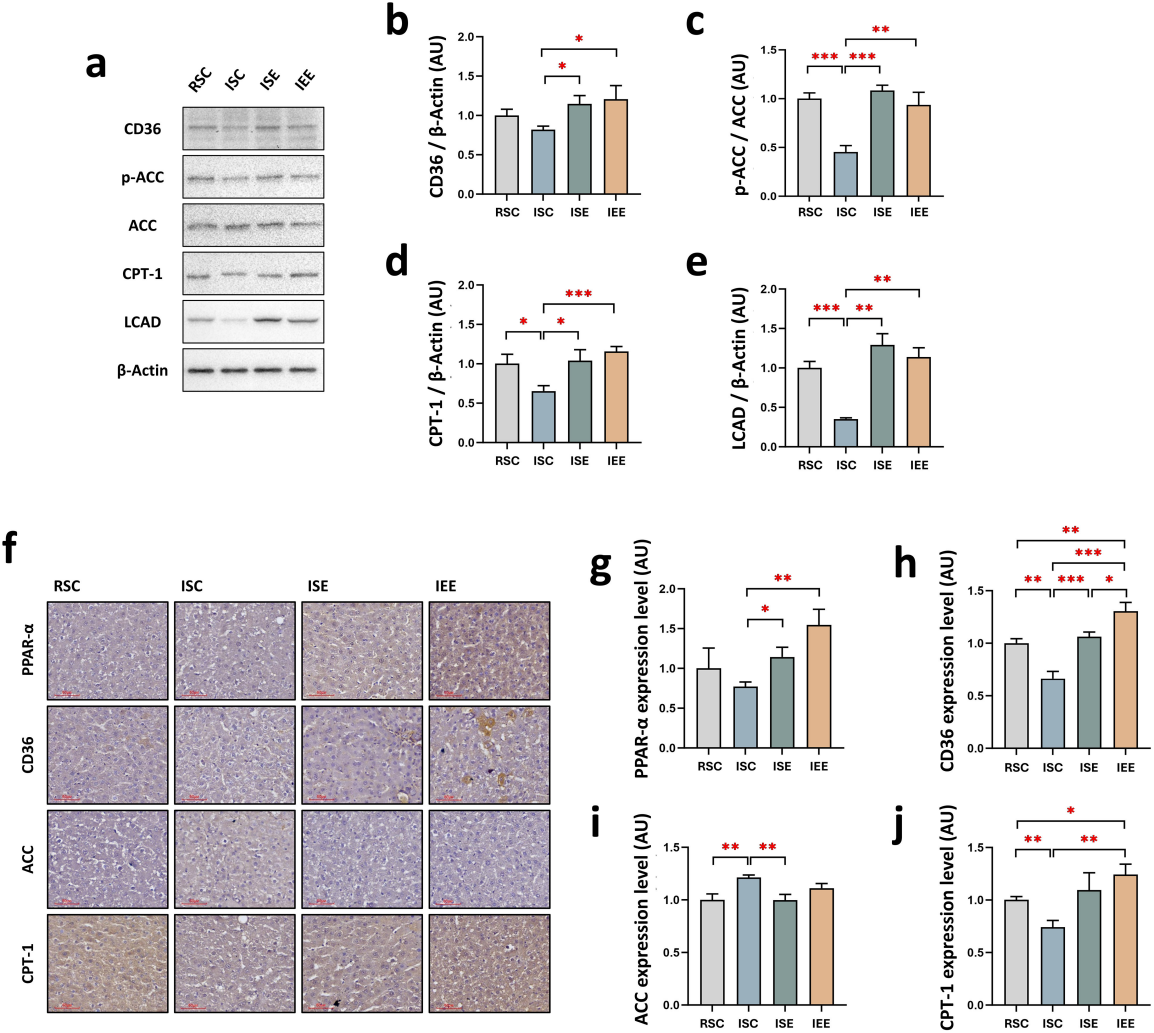
Effects of ETR on lipid metabolism–related factors in skeletal muscle and liver of rats under chronic circadian rhythm disruption. **(A)** Representative images of CD36, p-ACC, ACC, CPT-1, LCAD and β-actin in the gastrocnemius muscle as analyzed by Western blotting. **(B)** Expression levels of CD36 protein in the gastrocnemius muscle as analyzed by Western blotting. **(C)** Expression levels of p-ACC/ACC ratio in the gastrocnemius muscle as analyzed by Western blotting. **(D)** Expression levels of CPT-1 protein in the gastrocnemius muscle as analyzed by Western blotting. **(E)** Expression levels of LCAD protein in the gastrocnemius muscle as analyzed by Western blotting. **(F)** Representative IHC staining of PPAR-α, CD36, ACC, and CPT-1 in the liver. **(G)** Semi-quantitative analysis of PPAR-α immunoreactivity in the liver. **(H)** Semi-quantitative analysis of CD36 immunoreactivity in the liver. **(I)** Semi-quantitative analysis of ACC immunostaining intensity in the liver. **(J)** Semi-quantitative analysis of CPT-1 immunoreactivity in the liver. Data are presented as mean ± SD (n = 6 per group). Statistical analysis was performed using one-way ANOVA followed by Tukey’s *post hoc* test for multiple comparisons (P < 0.05; *P < 0.01; **P < 0.001; ns, not significant, P > 0.05).

To investigate the effects of CR disruption and ETR on lipid metabolism, the expression of lipid metabolism–related proteins in skeletal muscle and liver was analyzed (**Figure 3**).

CR disruption induced consistent dysregulation of lipid metabolism in both skeletal muscle and liver. In skeletal muscle, the ISC group exhibited significantly reduced expression of fatty acid oxidation–related proteins, including CPT-1 (**Figure 3D**; P < 0.05) and LCAD (**Figure 3E**; P < 0.001), along with a decreased p-ACC/ACC ratio (**Figure 3C**; P < 0.001).

In liver tissue, immunohistochemical staining suggested reduced CD36 and CPT-1 immunoreactivity in the ISC group (**Figures 3H, J**; P < 0.01), whereas ACC staining intensity appeared relatively increased (**Figure 3I**; P < 0.01).

ETR reversed these alterations. In skeletal muscle, both ISE and IEE groups exhibited increased expression of CD36, CPT-1, and LCAD (vs. ISC, P < 0.05), along with restoration of the p-ACC/ACC ratio (vs. ISC, P < 0.01) (**Figures 3A–D**).

In liver tissue, ETR partially restored the immunostaining patterns of PPAR-α, CD36, and CPT-1, while reducing ACC staining intensity compared with the ISC group (**Figures 3F–J**; P < 0.05). Notably, the IEE group exhibited relatively greater CD36 immunoreactivity than the ISE group (**Figure 3H**; P < 0.05), suggesting a more pronounced restoration of hepatic lipid metabolic profiles following prolonged exercise exposure.

### 3.5 Effects of different basal fitness levels on oxidative stress, adipogenesis and fibrosis in liver

**Figure 4:**
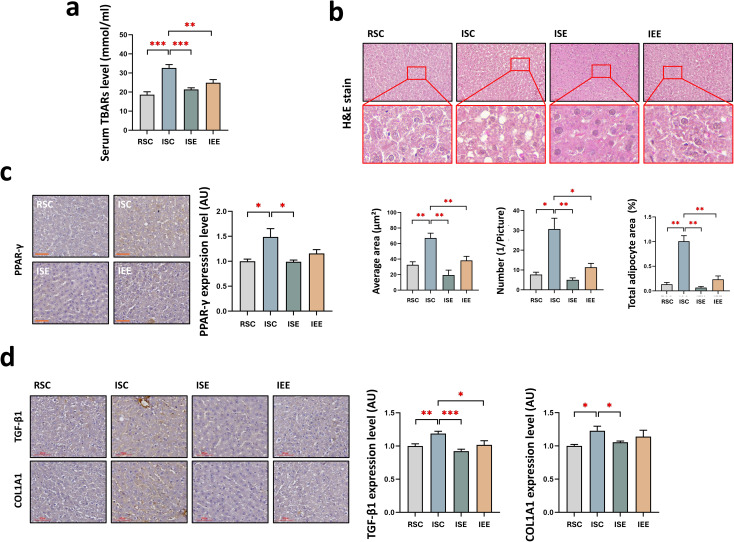
Effects of ETR on oxidative stress and liver lipogenesis and fibrosis-related markers in rats under chronic circadian rhythm disruption. **(A)** levels of TBARs in serum. **(B)** Representative H&E-stained images of liver and quantification of adipocyte average area, number, and total adipocyte area. **(C)** Representative IHC staining and semi-quantitative analysis of PPAR-γ immunoreactivity in the liver. **(D)** Representative IHC staining and semi-quantitative analysis of TGF-β1 and COL1A1 immunostaining intensity in the liver. Data are presented as mean ± SD (n = 6 per group). Statistical analysis was performed using one-way ANOVA followed by Tukey’s *post hoc* test for multiple comparisons (P < 0.05; *P < 0.01; **P < 0.001; ns, not significant, P > 0.05).

To determine the impact of CR disruption on liver metabolic health, oxidative stress, lipid accumulation, and fibrosis-related markers were evaluated (**Figure 4**).

CR disruption significantly increased systemic oxidative stress and promoted hepatic lipid accumulation and fibrotic remodeling. The ISC group exhibited significantly elevated serum TBARS levels compared with the other groups (**Figure 4A**; P < 0.01), indicating increased lipid peroxidation.

Histological analysis revealed that the ISC group showed increased adipocyte size, number, and total lipid area in the liver (**Figure 4B**; P < 0.05). In addition, enhanced PPAR-γ immunostaining was observed in the ISC group (**Figure 4C**), accompanied by increased immunostaining intensity of fibrosis-related markers, including TGF-β1 and COL1A1 (**Figure 4D**; P < 0.05).

ETR attenuated these alterations. Both ISE and IEE groups exhibited significantly reduced TBARS levels and improved hepatic histological features compared with the ISC group (**Figures 4A, B**; P < 0.05). Furthermore, ETR reduced the immunostaining intensity of PPAR-γ, TGF-β1, and COL1A1 compared with the ISC group (**Figures 4C, D**; P < 0.05), suggesting improved hepatic adipogenesis- and fibrosis-related histopathological alterations following exercise intervention. No significant differences were observed between the ISE and IEE groups for these parameters.

## 4 Discussion

The present study demonstrates that chronic CR disruption induces profound metabolic dysfunction in both skeletal muscle and liver, characterized by impaired clock protein expression (**Figure 1**), reduced mitochondrial biogenesis (**Figure 2**), disrupted substrate metabolism (**Figures 2**, **3**), increased oxidative stress (**Figure 4**), and enhanced liver lipid accumulation and fibrosis (**Figure 4**). Importantly, ETR effectively mitigates these alterations, with more pronounced improvements observed following prolonged exercise exposure and a higher training status.

CR disruption has been increasingly recognized as a critical factor contributing to metabolic disorders (19). In this study, the downregulation of BMAL1 and altered PER2 expression in skeletal muscle and liver confirm that prolonged light–dark cycle alternation disrupts peripheral circadian clocks (**Figure 1**). Given the central role of circadian regulators in coordinating metabolic pathways (20, 21), these disturbances likely underline the observed impairments in mitochondrial function and substrate utilization. Consistent with this notion, CR disruption markedly suppressed AMPK activation and PGC-1α signaling (**Figure 2A**), resulting in reduced mitochondrial biogenesis and oxidative capacity (1). The concomitant downregulation of GLUT4 and HK2 (**Figure 2C**), along with increased LDH expression (**Figure 2C**), suggests a shift in metabolic phenotype toward reduced oxidative metabolism (22, 23). These findings support the concept that circadian misalignment compromises metabolic flexibility in skeletal muscle. ETR effectively restored mitochondrial and metabolic function, potentially associated with enhanced AMPK activation and PGC-1α signaling (24, 25). Notably, the group with IEE exhibited greater improvements in mitochondrial protein expression and glucose transport capacity compared with the ISE, indicating that the duration of exercise exposure plays a critical role in enhancing metabolic adaptability under CR disruption. These findings highlight the importance of training history in determining the responsiveness to exercise interventions under chronically disrupted circadian conditions.

In addition to skeletal muscle, hepatic metabolism was markedly affected by CR disruption. The observed decrease in CD36 and CPT-1, along with increased ACC expression (**Figure 3**), indicates a shift toward lipid accumulation and reduced fatty acid oxidation. These alterations were accompanied by increased oxidative stress, lipid deposition, and upregulation of fibrosis-related markers, including TGF-β1 and COL1A1 (**Figure 4D**). Such changes are consistent with early pathological features associated with NAFLD and hepatic fibrotic remodeling (26, 27). ETR substantially attenuated these hepatic alterations. Both ISE and IEE groups showed reduced oxidative stress, improved liver histology, and decreased expression of adipogenic and fibrotic markers (**Figure 4**). Interestingly, no significant differences were observed between the ISE and IEE groups in oxidative stress and fibrosis-related outcomes. This suggests that the protective effects of endurance training on hepatic oxidative stress and fibrotic remodeling may be achieved even with relatively shorter intervention durations, whereas mitochondrial and metabolic adaptations appear to require more prolonged training exposure.

Taken together, these findings indicate that different metabolic systems exhibit distinct sensitivities to exercise duration under CR disruption. While mitochondrial function and substrate metabolism benefit from prolonged training exposure, hepatic oxidative stress and fibrosis-related pathways may respond more rapidly to exercise intervention. In addition, exercise timing may have contributed to the observed adaptations under chronic circadian rhythm disruption. Because endurance exercise was performed during the transition period between the rest and active phases, the interaction between exercise timing and circadian regulation cannot be excluded and may have influenced the metabolic responses observed in the present study.

From a translational perspective, circadian rhythm disruption is prevalent in modern working populations, including individuals engaged in both sedentary and physically active occupations (6). Although the present animal model does not directly replicate human occupational conditions, the observed differences between sedentary and exercise-trained states under CR disruption may provide insight into how habitual physical activity influences metabolic resilience in such populations.

Several limitations should be acknowledged. First, the relatively small sample size may have reduced the statistical power to detect subtle biological differences and may limit the generalizability of the findings. Second, direct measurements of aerobic capacity, such as VO_2_max, were not performed. Third, the underlying molecular mechanisms were not directly manipulated, and causal pathways require further investigation. Fourth, liver protein alterations were partially evaluated using DAB-based immunohistochemistry, which is inherently a semi-quantitative technique and may be influenced by tissue heterogeneity and staining variability. Therefore, these findings should be interpreted as supportive histological evidence rather than definitive quantitative measurements equivalent to Western blot analysis. Finally, only male rats were included in this study, which may limit the generalizability of the findings.

## 5 Conclusion

In summary, the present study demonstrates that endurance training effectively counteracts CR disruption–induced metabolic dysfunction in skeletal muscle and liver. Importantly, Prolonged training exposure enhances mitochondrial and metabolic adaptations, whereas protective effects on oxidative stress and fibrosis may occur even with shorter training duration. These findings highlight the importance of exercise as a practical strategy for mitigating metabolic disturbances associated with circadian misalignment.

## Data Availability

The original contributions presented in the study are included in the article/**Supplementary Material**. Further inquiries can be directed to the corresponding author.
