# Supplementary material for: Effects of endurance exercise on skeletal muscle and liver metabolic health in male rats with different fitness under chronic circadian rhythm disruption
Source: Front Endocrinol (Lausanne). 2026 Jul 15;17:1841754. doi: 10.3389/fendo.2026.1841754 (PMC13414137; doi:10.3389/fendo.2026.1841754)
Supplement: Supplementary file 1 [file SupplementaryFile1.docx]

Supplementary Material

## Supplementary Figures 1


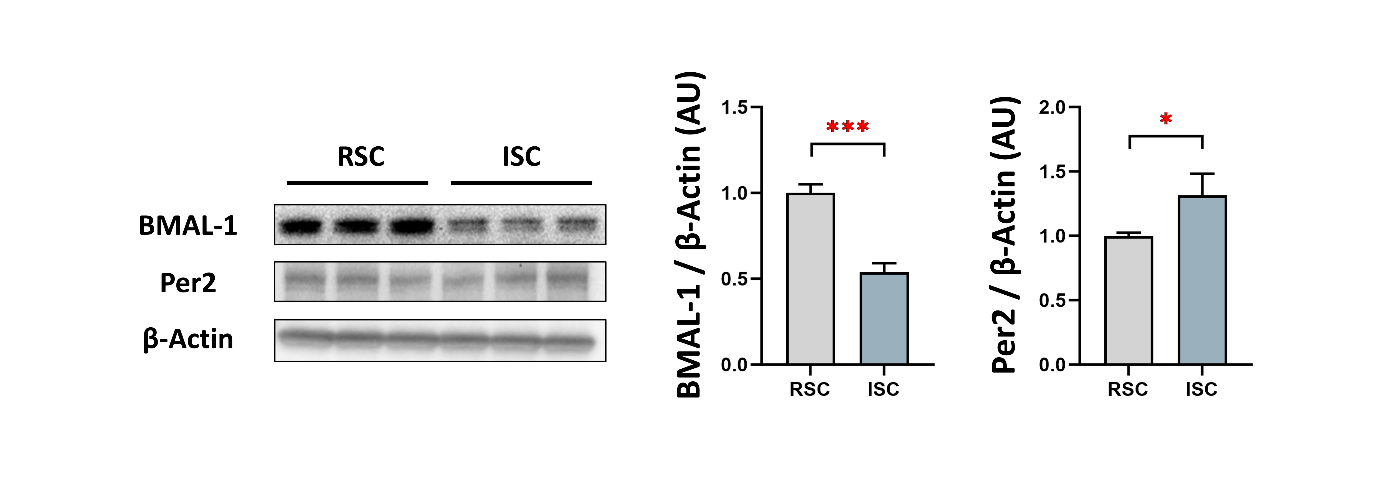


**Supplementary Figure 1.** Effects of 12-week circadian rhythm disruption on skeletal muscle clock factors in rats. Expression of BMAL1 and PER2 protein with representative western blot images. Data are presented as mean ± SD (n = 6 per group). Statistical analysis was assessed using an unpaired Student’s t-test (P < 0.05; *P < 0.01; **P < 0.001; ns, not significant, P > 0.05).

## Supplementary Figures 2


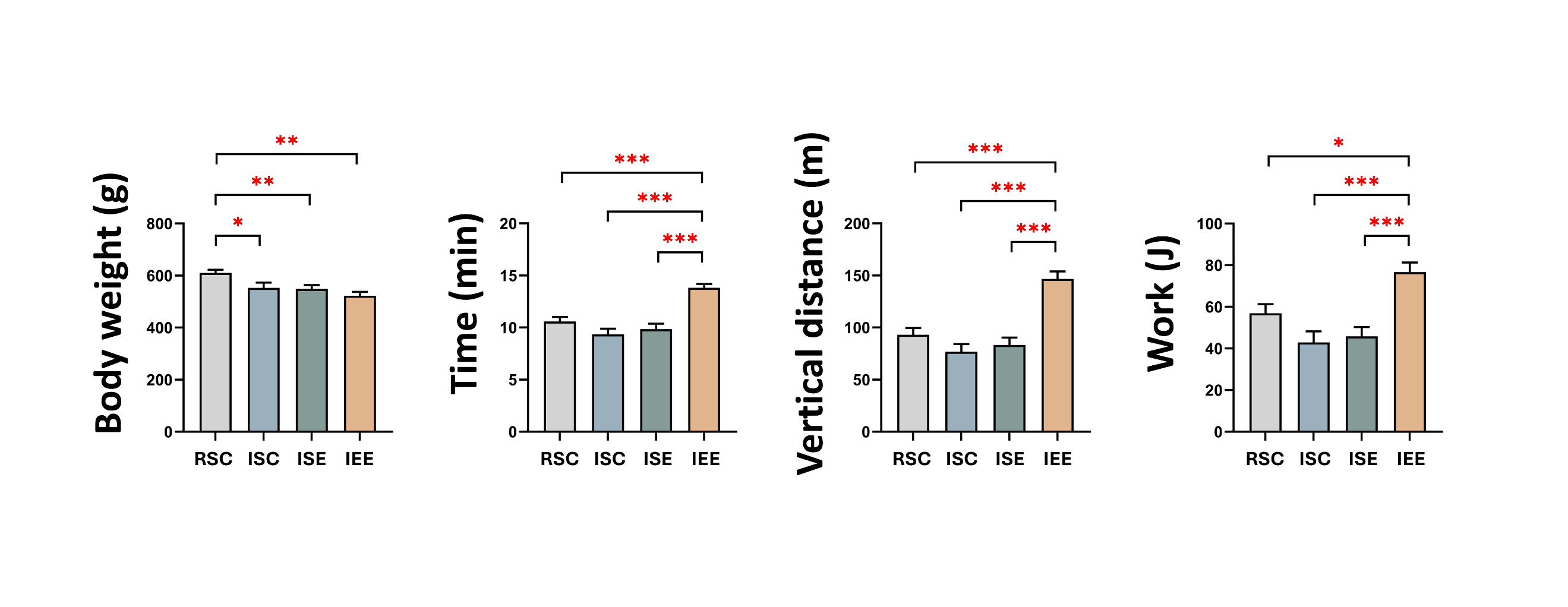


**Supplementary Figure 2.** Effects of 12-week circadian rhythm disruption on endurance exercise capacity test in rats. Body weight, running time (Time), vertical distance, and total work levels. Data are presented as mean ± SD (n = 6 per group). Data are presented as mean ± SD (n = 6 per group). Statistical analysis was performed using one-way ANOVA followed by Tukey’s post hoc test for multiple comparisons (P < 0.05; *P < 0.01; **P < 0.001; ns, not significant, P > 0.05).
